# An antipsychotic drug exerts anti-prion effects by altering the localization of the cellular prion protein

**DOI:** 10.1371/journal.pone.0182589

**Published:** 2017-08-07

**Authors:** Claudia Stincardini, Tania Massignan, Silvia Biggi, Saioa R. Elezgarai, Valeria Sangiovanni, Ilaria Vanni, Michael Pancher, Valentina Adami, Jorge Moreno, Matteo Stravalaci, Giulia Maietta, Marco Gobbi, Alessandro Negro, Jesús R. Requena, Joaquín Castilla, Romolo Nonno, Emiliano Biasini

**Affiliations:** 1 Dulbecco Telethon Laboratory of Prions and Amyloids, Centre for Integrative Biology (CIBIO), University of Trento, Trento, Italy; 2 Department of Molecular Biochemistry and Pharmacology, IRCCS-Istituto di Ricerche Farmacologiche Mario Negri, Milan, Italy; 3 Department of Food Safety and Veterinary Health, Istituto Superiore di Sanitá, Rome, Italy; 4 HTS Core Facility, Centre for Integrative Biology (CIBIO), University of Trento, Trento, Italy; 5 CIC bioGUNE, Parque tecnológico de Bizkaia, Derio; 6 Department of Biomedical Sciences, University of Padova, Padova, Italy; 7 CIMUS Biomedical Research Institute, University of Santiago de Compostela, Santiago de Compostela, Spain; 8 Department of Medical Sciences, University of Santiago de Compostela, Santiago de Compostela, Spain; 9 IKERBASQUE, Basque Foundation for Science, Bilbao, Bizkaia, Spain; 10 Department of Neuroscience, IRCCS-Istituto di Ricerche Farmacologiche Mario Negri, Milan, Italy; Deutsches Zentrum fur Neurodegenerative Erkrankungen, GERMANY

## Abstract

Prion diseases are neurodegenerative conditions characterized by the conformational conversion of the cellular prion protein (PrP^C^), an endogenous membrane glycoprotein of uncertain function, into PrP^Sc^, a pathological isoform that replicates by imposing its abnormal folding onto PrP^C^ molecules. A great deal of evidence supports the notion that PrP^C^ plays at least two roles in prion diseases, by acting as a substrate for PrP^Sc^ replication, and as a mediator of its toxicity. This conclusion was recently supported by data suggesting that PrP^C^ may transduce neurotoxic signals elicited by other disease-associated protein aggregates. Thus, PrP^C^ may represent a convenient pharmacological target for prion diseases, and possibly other neurodegenerative conditions. Here, we sought to characterize the activity of chlorpromazine (CPZ), an antipsychotic previously shown to inhibit prion replication by directly binding to PrP^C^. By employing biochemical and biophysical techniques, we provide direct experimental evidence indicating that CPZ does not bind PrP^C^ at biologically relevant concentrations. Instead, the compound exerts anti-prion effects by inducing the relocalization of PrP^C^ from the plasma membrane. Consistent with these findings, CPZ also inhibits the cytotoxic effects delivered by a PrP mutant. Interestingly, we found that the different pharmacological effects of CPZ could be mimicked by two inhibitors of the GTPase activity of dynamins, a class of proteins involved in the scission of newly formed membrane vesicles, and recently reported as potential pharmacological targets of CPZ. Collectively, our results redefine the mechanism by which CPZ exerts anti-prion effects, and support a primary role for dynamins in the membrane recycling of PrP^C^, as well as in the propagation of infectious prions.

## Introduction

There is a great need for the development of effective therapies for prion diseases, a class of fatal neurodegenerative conditions presenting motor dysfunction, dementia, and cerebral amyloidosis [1]. These disorders, which in human may occur sporadically (85%), genetically (10%), or horizontally transmitted (>5%), are characterized by the accumulation in nerve tissues of PrP^Sc^, an aggregated, protease-resistant and infectious isoform (prion) which replicates by inducing a conformational rearrangement of its endogenous counterpart (PrP^C^) into new PrP^Sc^ molecules [2]. Differences in the three-dimensional organization of PrP^Sc^ are believed to underline the biochemical and biological properties of the various prion strains found in mammals [3]. A variety of potential therapeutic approaches for prion diseases have been reported in the last three decades, with the vast majority of these efforts targeting the formation, replication, or stability of PrP^Sc^ [4]. A number of chemical classes have shown the ability to lower PrP^Sc^ in infected cell lines, and in some case prolong survival in mouse models [5]. Few of these molecules, such as quinacrine [6–9], pentosan polysulfate [10–13] and doxycycline [14,15], even reached the clinical phase. However, so far none of these approaches have shown efficacy in patients [16]. Moreover, several previous studies have raised concerns regarding the general concept of targeting PrP^Sc^. For example, while different prion strains showing wide structural heterogeneity may co-exist in the same host during prion infection [17], the vast majority of anti-prion compounds developed so far appear to be strain-specific [18]. In addition, few prion strains have also shown the ability to evolve in response to pharmacological treatments in cell cultures [19]. An additional confounding factor is related to the pathogenicity of PrP^Sc^, as this form seems to require functional PrP^C^ at the neuronal surface in order to exert its neurotoxic effects [20,21]. Collectively, these data suggest that PrP^Sc^ could be an inconvenient pharmacological target in prion diseases [22]. Targeting PrP^C^ could be an alternative therapeutic strategy [23,24]. In fact, compounds directed against PrP^C^ may produce the dual effect of interfering with the replication of multiple prion strains, and inhibit their neurotoxicity [25]. In support of this notion, several approaches aimed at silencing PrP^C^ have shown strong potentials to alter prion pathogenesis. For example, rescue of memory performance and a remarkable extension of lifespan in prion-infected mice were obtained by a single injection in the hippocampus of lentiviral-encoded short hairpin RNAs against PrP^C^ [26]. Moreover, several polyanionic polymers and sulfated glycans have been shown to inhibit prion replication in various experimental models by removing PrP^C^ from the plasma membrane [27,28]. Some phenothiazine derivatives, including CPZ, were previously shown to directly bind PrP^C^, inducing an intra-molecular conformational rearrangement that could explain the ability of these compounds to inhibit the replication of different prion strains in cell cultures [6,7,29,30]. In this manuscript, we employed various biochemical, biophysical and cell-based techniques to further characterize the mechanism of action of CPZ.

## Results

### CPZ inhibits prion replication in cells but not in vitro

**Fig 1:**
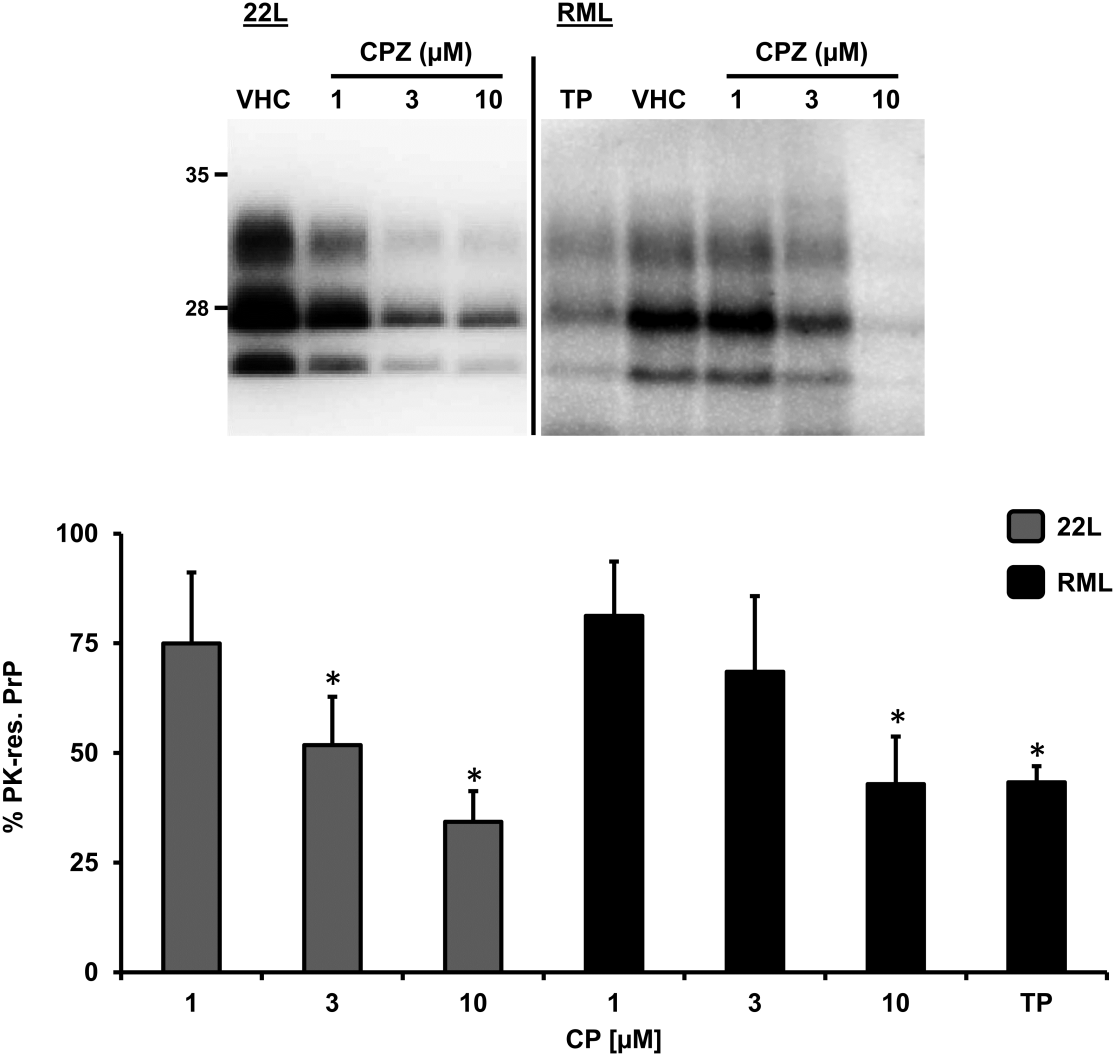
CPZ inhibits prion replication in cells. N2a cells chronically infected with the 22L or RML prion strains were incubated with increasing concentrations (indicated) of CPZ, TP (10 μM), or vehicle (VHC) for 72h. The level of PrP^Sc^ in cell lysates was estimated by detecting the amount of PK-resistant PrP by Western blotting, using anti-PrP^C^ antibody D18. Signals from at least three independent experiments (n>3) were quantified by densitometric analysis of gel blots, normalized on the total amount of proteins (obtained by Ponceau staining) in PK-untreated replicates, and expressed in the bar graph as mean percentage (%) of the signal in vehicle-treated cells (± standard error). Statistical differences (*) between CPZ and vehicle control were estimated by Student *t*-test: for the 22L strain, [1 μM], *p* = 0.209; [3 μM], *p* = 0.074; [10 μM], *p* = 0.041; for RML strain, [1 μM] *p* = 0,274; [3 μM] *p* = 0,123; [10 μM] *p* = 0,0451. Estimated IC_50_ values for CPZ were as follow: for the 22L strain, 4.28 ± 1.67 μM; for the RML strain, 7.32 ± 1.92.

In order to confirm previously reported anti-prion effects of CPZ, we exposed N2a cells chronically infected with either 22L or RML mouse prion strains, to different concentrations of CPZ (1–10 μM), the porphyrin Fe(III)-TMPyP (TP, 10 μM) or vehicle (VHC) control for 72h. Consistent with previous studies, treatment with CPZ caused a dose-dependent decrease of proteinase K (PK)-resistant PrP levels, as detected by Western blotting (Fig 1). The estimated inhibitory concentration at 50% (IC_50_) for CPZ in 22L- or RML-infected N2a cells were fully compatible with previously published data (~3 μM) [6].

**Fig 2:**
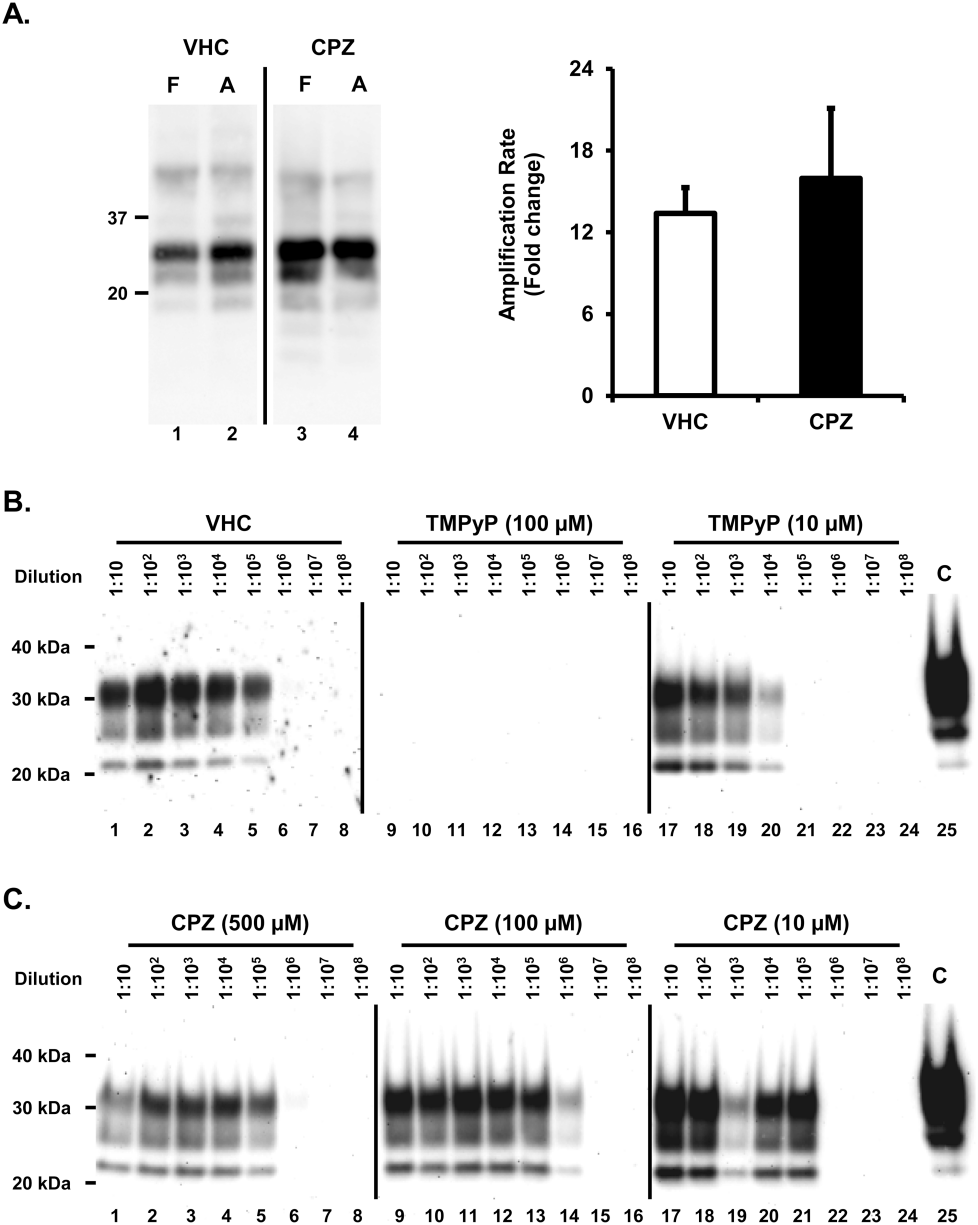
CPZ does not affect prion amplification in vitro. **A.** Brain homogenates from terminally ill voles infected with an Italian vole-adapted scrapie strain were diluted 1:10 (F, lanes 1 and 3) or 1:100 (A, lanes 2 and 4) in PMCA substrate in presence of vehicle (VHC, lanes 1–2) or 500 μM CPZ (lanes 3 and 4). Samples diluted 1:100 were subjected to a single PMCA round, while those diluted 1:10 were kept frozen and used to determine the amplification factor. Samples were PK-digested and analyzed by Western Blotting with antibody SAF84. The graph illustrates mean amplification factors (± standard error) obtained with CPZ or vehicle alone, from three independent experiments (n = 3). We detected no statistical differences between CPZ and vehicle control. **B.** A 50 μl aliquot of 1% Tg338 brain homogenate, seeded with different dilutions of scrapie (1:10–1:108) were mixed with 0.5 μl of DMSO, TMPyP (used as positive control) or CPZ diluted in DMSO (final concentrations as shown in the picture) and subjected to a unique 24 h round of standard PMCA. Amplified samples were digested with 200 μg/ml of proteinase K (PK) and analyzed by western blotting using monoclonal antibody SAF83 (1:400). While TMPyP exhibited a potent inhibitory activity toward prion amplification (estimated to be at least 10^5^ fold at 100 μM and 10−10^2^ fold at 10 μM), CPZ did not show any inhibitory effect even at the highest concentration (500 μM). C: Normal, untreated brain homogenate. The results are representative of three independent replicates (n = 3).

CPZ was recently reported to bind PrP^C^ [30]. Since PrP^C^ is the common substrate of any prion replication reaction, a compound binding to its native conformation could theoretically show inhibitory effects towards multiple prion strains, both in cells and in vitro. In a recent study, we provided such evidence for the cationic tetrapyrrole Fe(III)-TMPyP [25]. Here, we employed the protein misfolding cyclic amplification (PMCA) reaction to test whether CPZ acts in a similar fashion. PMCA is a widely used methodology to propagate prions in vitro, based on the principle of mixing a large amount of PrP^C^ substrate molecules with small amounts of brain- or cell-derived PrP^Sc^ particles, subjecting the mixture to consecutive cycles of sonication and incubation [31,32]. We recently described a modified PMCA protocol to rapidly test the potential inhibitory effects of small molecules [25,33]. In this assay, brain homogenates of bank voles homozygous for methionine at codon 109 (Bv109M) are used as substrate for the conversion of bank vole-adapted sheep scrapie, diluted 1:100. In absence of inhibitors, this PMCA protocol produces a ≥10 fold prion amplification in 16 hours. As previously observed, micromolar amounts of Fe(III)-TMPyP strongly inhibited the replication of PrP^Sc^ (data not shown). Conversely, CPZ produced no inhibitory effects even at high concentrations (100–500 μM), with prion amplification rates similar (~12 folds) in CPZ-treated and VHC-treated samples (Fig 2A). To further substantiate these results, we employed an alternative PMCA protocol (Fig 2B and 2C). In this case, the PMCA reaction was performed by mixing different dilutions of scrapie prions (Dawson isolate, from 1:10 to 1:10^8^), with brain lysates derived from transgenic (Tg) mice expressing ovine PrP (called Tg338), and then adding Fe(III)-TMPyP (100 or 500 μM), CPZ (10, 100 or 500 μM) or VHC (control). Samples were then subjected to a single, 24h-long round of PMCA. PrP^Sc^ levels were then estimated by detecting PK-resistant PrP molecules by Western blotting. In control samples, we observed robust PrP^Sc^ amplification up to the 10^5^ dilution, while in presence of Fe(III)-TMPyP such amplification was drastically reduced (10 μM) or completely abolished (100 μM, Fig 2B). Conversely, CPZ showed no inhibitory effects even at the highest concentration (500 μM, Fig 2C). We concluded that the compound exhibited no inhibitory activity toward the in vitro amplification of PrP^Sc^. Collectively, these results indicate that CPZ blocks prion replication in cells but not in vitro, suggesting that its mode of action might differ from the one reported for Fe(III)-TMPyP.

### CPZ suppresses the drug-hypersensitizing effect of a mutant PrP

**Fig 3:**
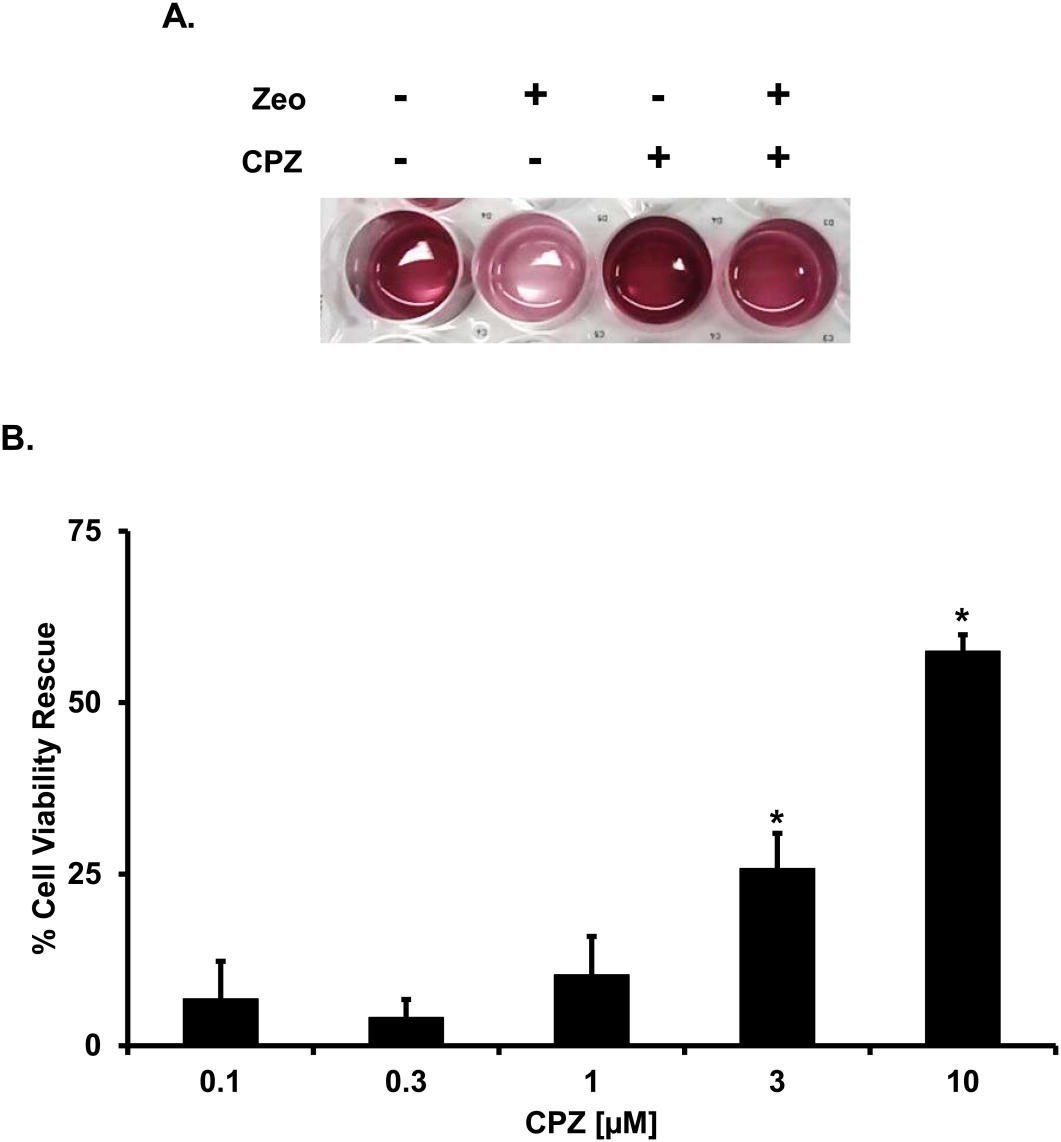
CPZ suppresses the drug-hypersensitizing effect of ΔCR PrP. **A.** The DBCA was employed to evaluate the anti-ΔCR PrP effects of CPZ. Stably transfected ΔCR HEK293 cells carrying the hygromycin B resistance cassette were plated in 24-well plates and incubated in medium containing 500 μg/mL of Zeocin, for 48h at 37°C. The picture shows an example of wells after MTT assay (CPZ 10 μM). **B.** The bar graph illustrates the quantification of the dose-dependent rescuing effect of CPZ. Mean values were obtained from a minimum of 6 independent experiments (n = 6), and expressed as percentage of cell viability rescue, using the following equation: R = (T-Z)/(U-Z) (R: rescuing effect; T: cell viability in CPZ-treated samples; Z: cell viability in zeocin-treated samples; U: cell viability in untreated samples). Statistically-significant differences (*) between CPZ-treated and untreated cells were estimated by Student *t*-test: [0.1 μM], *p* = 0.12381; [0.3 μM], *p* = 0.10209; [1 μM], *p* = 0.05764; [3 μM], *p* = 0.00109; [10 μM], *p* = 4.35 x 10^−9^.

PrP^C^ molecules carrying artificial deletions (Δ) or disease-associated point mutations in the conserved central region (CR) have been shown to confer hypersensitivity to several cationic antibiotics (e.g. bleomycin/phleomycin analogues such as Zeocin, or aminoglycosides such as G418 or hygromycin) in various cultured cells and primary neurons [34–36]. This detrimental effect can be easily monitored by a previously described drug-based cellular assay (DBCA) [37,38]. The DBCA can be used to test the activity of potential inhibitors of mutant PrP toxicity. For example, we have recently shown that Fe(III)-TMPyP inhibits the drug-hypersensitizing effects of one of the most toxic PrP mutants reported so far, carrying a 20 amino acids deletion in the central region (Δ105–125, called ΔCR) [25]. Here, we employed the DBCA to evaluate the effects of CPZ against ΔCR toxicity (Fig 3). HEK293 cells stably transfected with ΔCR PrP were exposed to Zeocin (500 μg/mL) for 48h, in presence (0.1–10 μM) or absence of CPZ. As expected, Zeocin-treated cells showed a strong reduction in viability (< 70%), as assayed by MTT. Conversely, co-treatment with CPZ caused a dose-dependent rescue of antibiotic-induced cell death, with IC_50_ values similar to those observed in prion-infected cells (IC_50_ = 5.03 ± 2.97 μM). CPZ was not toxic to control cells expressing wild type (WT) PrP in the same experimental conditions (S1A Fig). These results demonstrate that CPZ inhibits the drug-hypersensitizing effects of ΔCR PrP.

### CPZ is a weak ligand of PrP^C^

**Fig 4:**
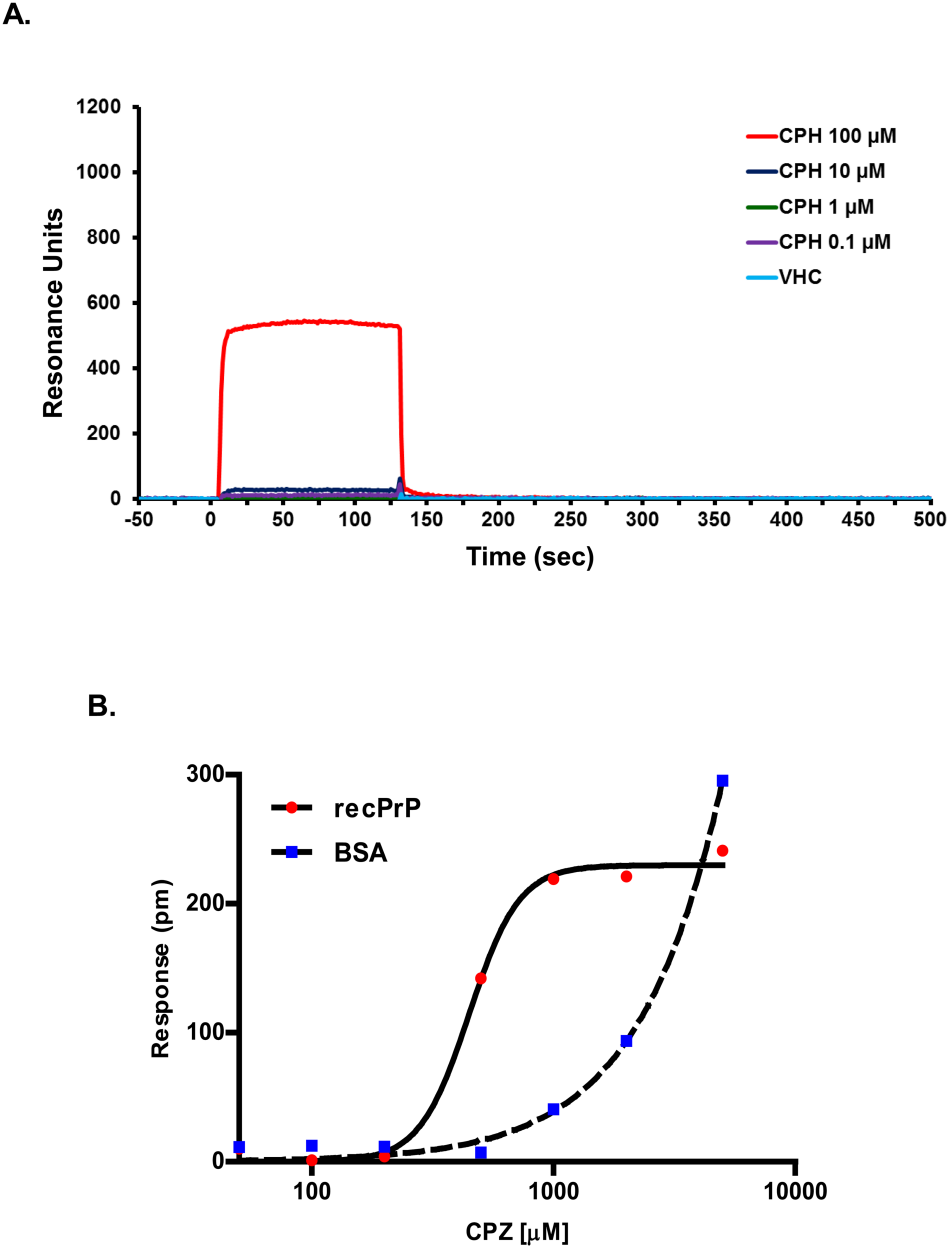
CPZ is a weak ligand of PrP^C^. **A.** The interaction of CPZ with recombinant PrP^C^ was evaluated by SPR. Starting at time 0, the indicated concentrations of CPZ were injected for 130 sec over sensor chip surfaces (GL-H chip, Bio-Rad) on which 16.000 resonance units (RU) of full-length, mouse recombinant PrP^C^ had previously been captured by amine coupling. The chip was then washed with PBST buffer alone to monitor ligand dissociation. Sensorgrams show CPZ binding in RU. The data were obtained by subtracting the reference channels. No reliable fitting was obtained for any of the curves, a fact that undermined the calculation of the kinetic constants for the interaction. **B.** CPZ-PrP^C^ interaction by DMR. Different concentrations of CPZ were added to label-free microplate well surfaces (EnSpire-LFB HS microplate, Perkin Elmer) on which full-length human recombinant PrP^C^ or BSA had previously been immobilized. Measurements were performed before (baseline) and after (final) adding the compound. The response (pm) was obtained subtracting the baseline output to the final output signals. The output signal for each well was obtained by subtracting the signal of the protein-coated reference area to the signal of uncoated area. The CPZ signals (red dots) were fitted (black line) to a sigmoidal function using a 4 parameter logistic (4PL) non-linear regression model; *R*^*2*^ = 0.99; *p* = 0.00061.

A previous study reported that the binding of phenothiazine derivatives, including promazine and CPZ, to PrP^C^ induces an allosteric reorganization of the N-terminal tail around the C-terminal, globular domain of the protein [30]. This model could explain the observed effects of CPZ against prion replication, as well as ΔCR PrP toxicity [23]. However, binding of CPZ to PrP^C^ was observed by X-ray crystallography and NMR, two techniques that require relatively high (mM) concentrations of compound, while a precise affinity value for the interaction was not reported. Since CPZ inhibits prion replication and mutant PrP toxicity in the low micromolar range, we sought to confirm binding of this compound to PrP^C^ at similar concentrations. First, we employed surface plasmon resonance (SPR), a biophysical technique capable of monitoring association and dissociation of two molecules in a kinetic fashion (Fig 4A). SPR was recently used to confirm the binding of Fe(III)-TMPyP to PrP^C^ [25]. We immobilized recombinant, mouse PrP^C^ on the surface of an SPR chip (immobilization was confirmed with anti-PrP antibody 6D11 flowed over the surface). Next, we injected different concentrations of CPZ (0.1–100 μM), and detected association and dissociation over the course of 500 seconds. Specific binding was almost exclusively detected at 100 μM, and was characterized by a very fast association and dissociation, whose steepness prevented a reliable estimation of corresponding rate constants. However, since SPR signals at the equilibrium are proportional to the concentration of the analyte, these data indicate that the affinity constant (K_D_) of CPZ for PrP^C^ is probably higher than 100 μM. Unfortunately, the compound showed large, non-specific interactions with the sensor surface at higher concentrations (not shown), preventing further analyses. Thus, we turned to another label-free, biophysical technique, called dynamic mass redistribution (DMR), which was previously used to define the affinity of Fe(III)-TMPyP to PrP^C^ at the equilibrium (Fig 4B) [25]. We immobilized recombinant human PrP^C^ or bovine serum albumin (BSA) on the surface of a 384-well, label-free microplate by amine-coupling chemistry. Different concentrations of CPZ (10–5,000 μM) were added, and binding was detected after a 30 min incubation. Empty DMR surfaces (built in each microplate well) and buffer injections were used to normalize the signals. We observed a dose-dependent binding of CPZ to PrP^C^ in the concentration range of ~200–1,000 μM, as indicated by the typical sigmoidal distribution of the data. Binding appeared to saturate at higher concentrations. These results allowed us to estimate the affinity of CPZ for PrP^C^ in the high micromolar range (K_D_ = 421 μM). Importantly, CPZ showed binding to BSA in a similar concentration range, calling into question the binding specificity of the compound. We concluded that CPZ interacts with PrP^C^ with a relatively weak affinity, and probably with low specificity.

### CPZ decreases the level of PrP^C^ at the cell surface

**Fig 5:**
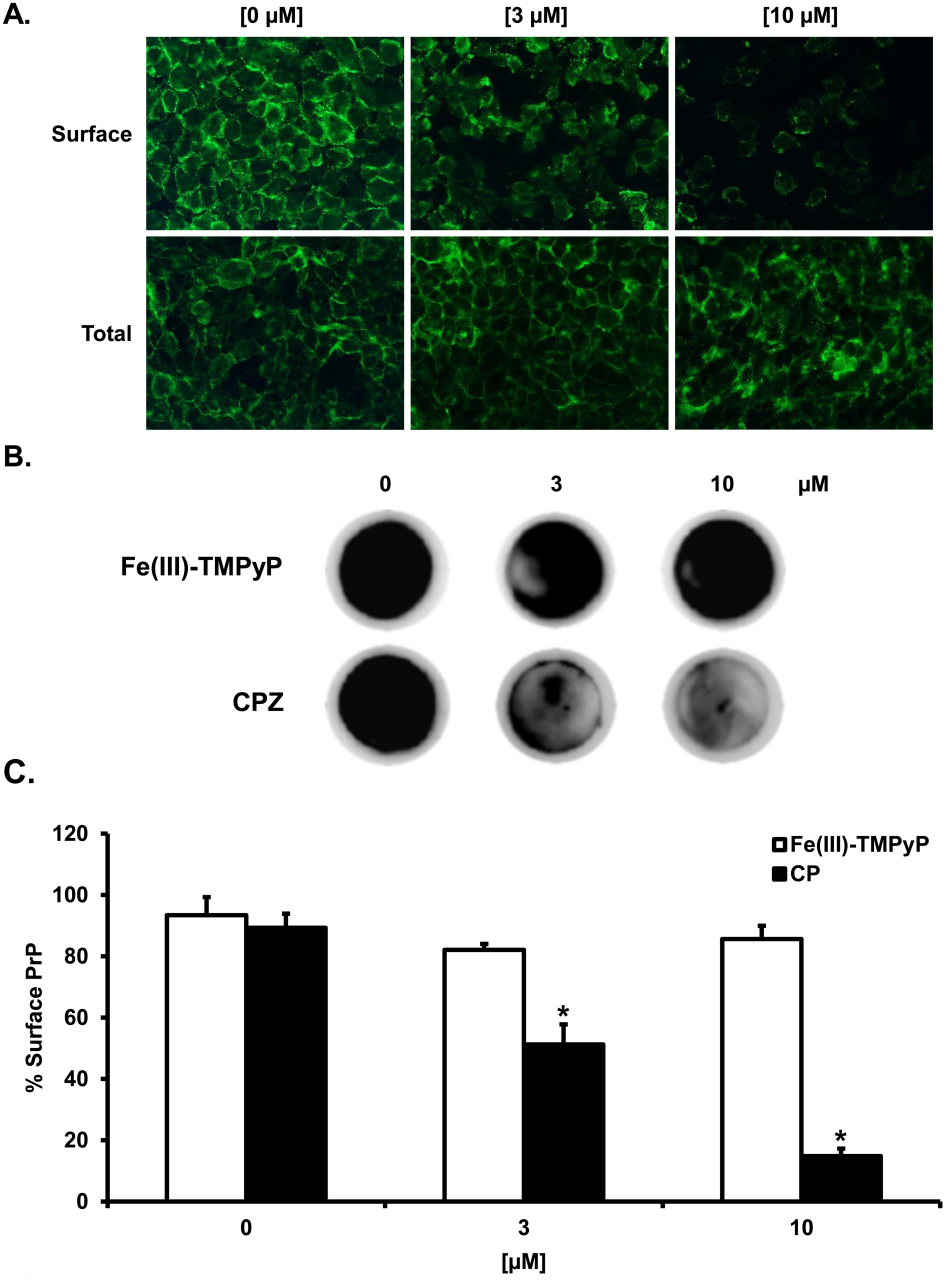
CPZ alters the cell surface localization of PrP^C^. **A.** Cells were seeded on glass coverslips and grown for 24 h to ~60% confluence. For surface staining of PrP, cells were first incubated at 4°C with antibody D18 diluted, then fixed with paraformaldehyde and incubated with fluorescently-labelled secondary antibody. For total PrP staining, cells were permeabilized with Triton X-100, fixed with paraformaldehyde, and then incubated with primary and secondary antibodies. Coverslips were mounted with Fluor-save Reagent (Calbiochem), and analyzed with a Zeiss Imager M2 microscope. **B.** N2a cells stably expressing mouse WT PrP^C^ were grown to confluence on glass coverslips, and treated with the indicated concentrations of Fe(III)-TMPyP or CPZ for 24h. For detection of surface PrP^C^ (SC#1, shown in the picture), coverslips were incubated in ice with antibody 6D11 (this step was omitted for detection of total PrP^C^, not shown). Coverslips were blotted on a nitrocellulose membrane soaked in lysis buffer, and incubated with horseradish peroxidase-conjugated secondary antibody. For detection of total PrP^C^, cell blots were incubated with the primary and secondary antibodies. The PrP^C^ signal was revealed by enhanced chemiluminescence. **C.** PrP^C^ signal was quantitated by densitometry. The bar graph shows the % ratio of surface to total PrP^C^. Each bar represents the mean (± standard error) of three independent experiments (n = 3). Statistically-significant differences (*), estimated by Student *t*-test, between CPZ-treated and untreated cells were as follow: [3 μM], *p* = 0.0058; [10 μM], *p* = 0.00034.

Our data indicate that CPZ is active in cells against prion replication and mutant PrP toxicity at concentrations approximately one-hundred times lower than its affinity for PrP^C^. This conclusion implies that most likely the compound does not exert its effect by interacting directly with PrP^C^. In addition to its antipsychotic effects, CPZ is known to inhibit clathrin-mediated endocytosis (CME) by a mechanism not completely understood [39,40]. Since it has been reported that CME plays a role in the recycling of PrP^C^ from the plasma membrane [41–43], we decided to evaluate whether CPZ may exert anti-prion effects by altering the levels of PrP^C^ at the cell surface. In order to test this hypothesis, we treated HEK293 cells stably expressing WT PrP with biologically active concentrations of CPZ (3 and 10 μM), and detected the amount of PrP^C^ in permeabilized (total PrP^C^) or non-permeabilized (surface PrP^C^) cells using an anti-PrP antibody (D18). We detected an evident decrease of PrP^C^ signal at the cell surface upon treatment with CPZ. In order to corroborate these data, we employed a cell blot technique previously used to quantify the cell surface levels of misfolded mutants of PrP [44]. The technique is based on the concept of labeling PrP^C^ with specific antibodies in intact cells. Since antibodies are usually unable to cross the cell membrane, the only population of antibody-reacting PrP^C^ molecules would be the one expressed at the cell surface. N2a cells stably expressing mouse WT PrP^C^ were grown to confluence on glass coverslips. For detection of surface PrP^C^, we incubated a first set of coverslips (SC#1) on ice with antibody 6D11. This step was avoided for a second set of coverslips (SC#2). Both sets (SC#1 & #2) were then blotted directly onto a nitrocellulose membrane soaked in lysis buffer. In order to detect surface-exposed PrP^C^, the membrane corresponding to SC#1 was directly incubated with an horseradish peroxidase (HRP)-conjugated secondary antibody. Conversely, total PrP^C^ was revealed by incubating the membrane containing SC#2 with both primary (6D11) and secondary antibodies. The relative amount of cell surface PrP^C^ was then calculated by dividing the signal of SC#1 (surface PrP^C^) for the signal obtained from SC#2 (total PrP^C^). In normal conditions, the signal of surface PrP^C^ equals almost entirely the amount of total PrP^C^, indicating that the vast majority of molecules are expressed at the cell surface. Conversely, pre-incubation for 24h with two different concentrations of CPZ (3–10 μM) caused a dose-dependent decrease in the percentage of surface-exposed PrP^C^ (Fig 5). Consistent with previous data [25], this effect was not observed when cells were treated with Fe(III)-TMPyP. Of note, the active concentration of CPZ in this assay was very consistent with the IC_50_ values observed in prion-infected cells and in the DBCA.

**Fig 6:**
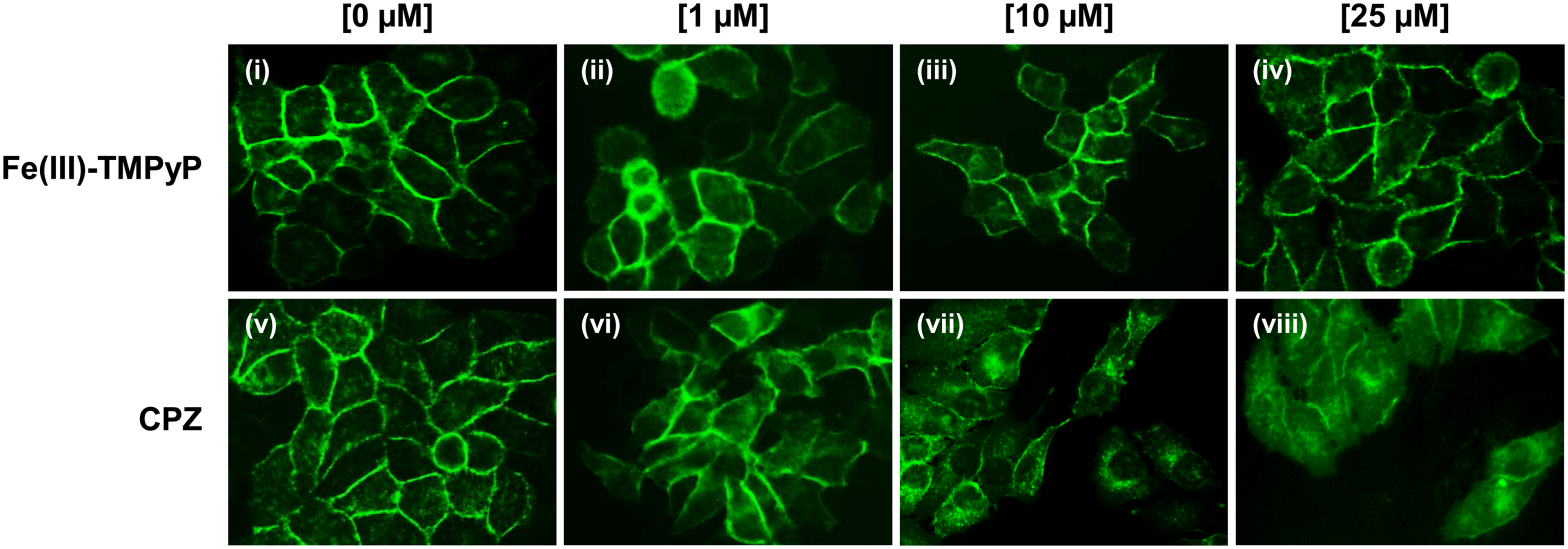
CPZ changes the cell surface distribution of EGFP-PrP^C^. HEK293 cells stably expressing EGFP-PrP^C^ were grown to ~60% confluence on glass coverslips, and then treated with the indicated concentrations of CPZ or Fe(III)-TMPyP for 24h. After fixation and washing, the intrinsic green signal of EGFP-PrP^C^ was acquired with an inverted microscope coupled with a high-resolution camera equipped with a 488 nm excitation filter.

Next, we sought to confirm these results using a completely different experimental paradigm (Fig 6). In order to directly observe PrP^C^ localization, we stably transfected HEK293 cells with a PrP^C^ form tagged with a monomerized EGFP molecule at its N-terminus (EGFP-PrP^C^). In control conditions, EGFP-PrP^C^ localizes almost entirely at the plasma membrane, giving rise to a typical “honeycomb-like” staining of the cell surface. As previously reported [25], EGFP-PrP^C^ localization was not altered by incubation for 24h with different concentrations of Fe(III)-TMPyP (1–25 μM). Conversely, treatment with CPZ (1–25 μM) caused a robust, dose-dependent redistribution of EGFP-PrP^C^ from the plasma membrane to intracellular compartments (Fig 6). At the concentration of 10 μM CPZ, such re-localization effect was already evident at time points as early as 8h (not shown), while only at the highest concentration (25 μM) after 48h incubation the compound showed detectable cytotoxicity (Fig 6C and S1B Fig). Collectively, these results demonstrate that CPZ is capable of decreasing the amount of PrP^C^ at the cell surface, an effect exerted in the same range of concentrations at which the molecule shows inhibitory activity toward prion replication and mutant PrP toxicity.

**Fig 7:**
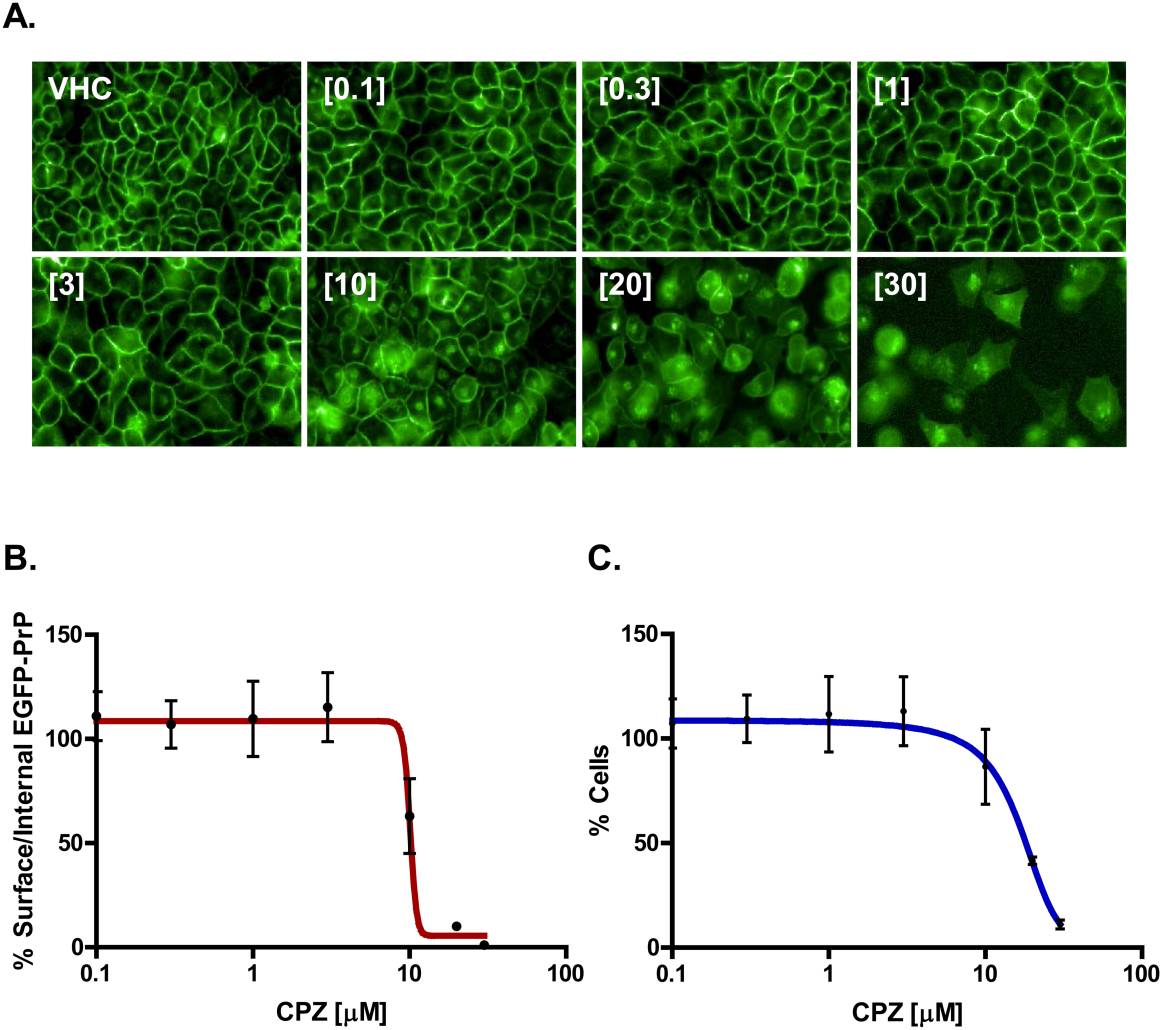
Semi-automatic detection of EGFP-PrP^C^ distribution upon CPZ treatment. **A.** HEK293 cells stably expressing EGFP-PrP^C^ were grown to ~80% confluence on 384-well plates, and then incubated with the indicated concentration of CPZ for 24h. An Operetta High-Content Imaging System was then employed to calculate the cell surface vs intracellular amount of EGFP-PrP^C^. **B.** The graph shows the mean percentage of cells (± standard deviation) showing a ratio of membrane vs intracellular EGFP-PrP^C^ signal higher than 1.5, after treatment with raising concentrations (indicated) of CPZ. **C.** The graph shows the mean percentage (± standard deviation) of the total number of nuclei detected in each well by Hoechst staining, after treatment with raising concentrations (indicated) of CPZ.

In order to corroborate these observations, we employed a semi-automatic, high-content imaging system (Operetta, Perkin Elmer), and quantified the ability CPZ to alter the cell surface localization of EGFP-PrP^C^ (Fig 7 and S7 Fig). HEK293 cells stably expressing EGFP-PrP^C^ were seeded on 384-well plates, grown to ~80% confluence, and then exposed for 24h to different concentrations of the compound (0.1–30 μM). We then measured two different parameters: (i) the number of cells showing a ratio <1.5 between surface and internal EGFP-PrP^C^ signal (Fig 7B); (ii) the number of cell nuclei (stained by Hoechst) (Fig 7C). These analyses confirmed that CPZ induces an evident relocalization of EGFP-PrP^C^ from the cell surface, at concentrations between 3 and 10 μM. Above 10 μM, the molecule started to show cytotoxicity.

### Dynamin inhibitors induce the relocalization of PrP^C^ from the cell surface

**Fig 8:**
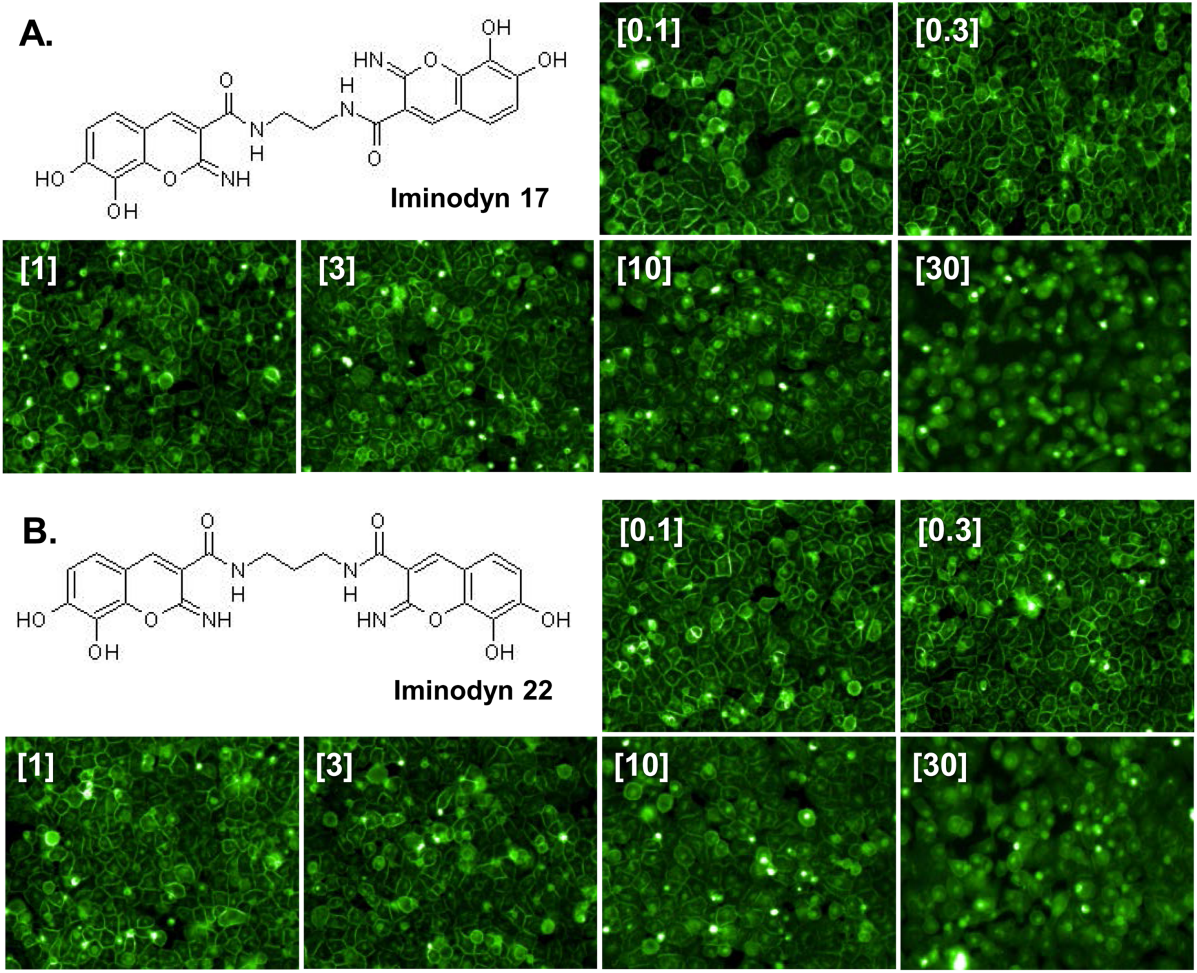
Inhibitors of the GTPase domain of dynamin I and II induce the redistribution of PrP^C^ from the cell surface. **A-B**. High-content analysis of HEK293 cells stably expressing EGFP-PrP^C^ after a 24h treatment with two dynamin inhibitors (at the indicated concentrations). The chemical structure of each compound, and representative images are shown. **C-D.** Graphs show the mean percentage of cells (± standard deviation) showing a ratio of surface vs intracellular EGFP-PrP^C^ signal higher than 1.5, after treatment with raising concentrations of each molecule. **E-F.** Toxicity profile of Iminodyn-17 and Iminodyn-22. The graphs show the mean percentage (± standard deviation) of the total number of cell nuclei in Iminodyn-17- or Iminodyn-22-treated cells, as detected by Hoechst staining.

A recent study found that the ability of several phenothiazine-derived antipsychotics to inhibit CME in cells is dependent upon the GTPase activity of dynamins [39]. Interestingly, eight different phenothiazines, including CPZ, were shown to inhibit dynamin I and II in the low micromolar range (1–12 μM), suggesting that dynamins might be the pharmacological target through which phenothiazines inhibit CME. Since CPZ induces the redistribution of PrP^C^ from the cell surface in the same concentration range (1–10 μM), we speculated that the effect on PrP^C^ localization could be mimicked by inhibiting dynamins. In order to test this hypothesis, we checked the ability of different dynamin inhibitors to alter the cell surface localization of EGFP-PrP^C^. These included molecules known to target the lipid-binding domain of dynamins, as well as compounds reported to target the GTPase domain. Molecules directed against the lipid-binding domain of dynamin I and II showed no effect on EGFP-PrP^C^ localization (Pro-Myristic Acid, S2 Fig), or high cytotoxicity even at low μM concentrations (MiTMABTM and OcTMABTM, S3 and S4 Figs). Conversely, four compounds (Dynole-31-2, Dynole-34-2, S5 and S6 Figs; Iminodyn-17 and Iminodyn-22, Fig 8A–8D) directed against the GTPase domain of dynamin I and II induced the relocalization of EGFP-PrP^C^ from the cell surface, with two of these molecules also showing relatively low cytotoxicity (Fig 8E and 8F).

### The anti-prion effects of CPZ could be mimicked by dynamin inhibitors

**Fig 9:**
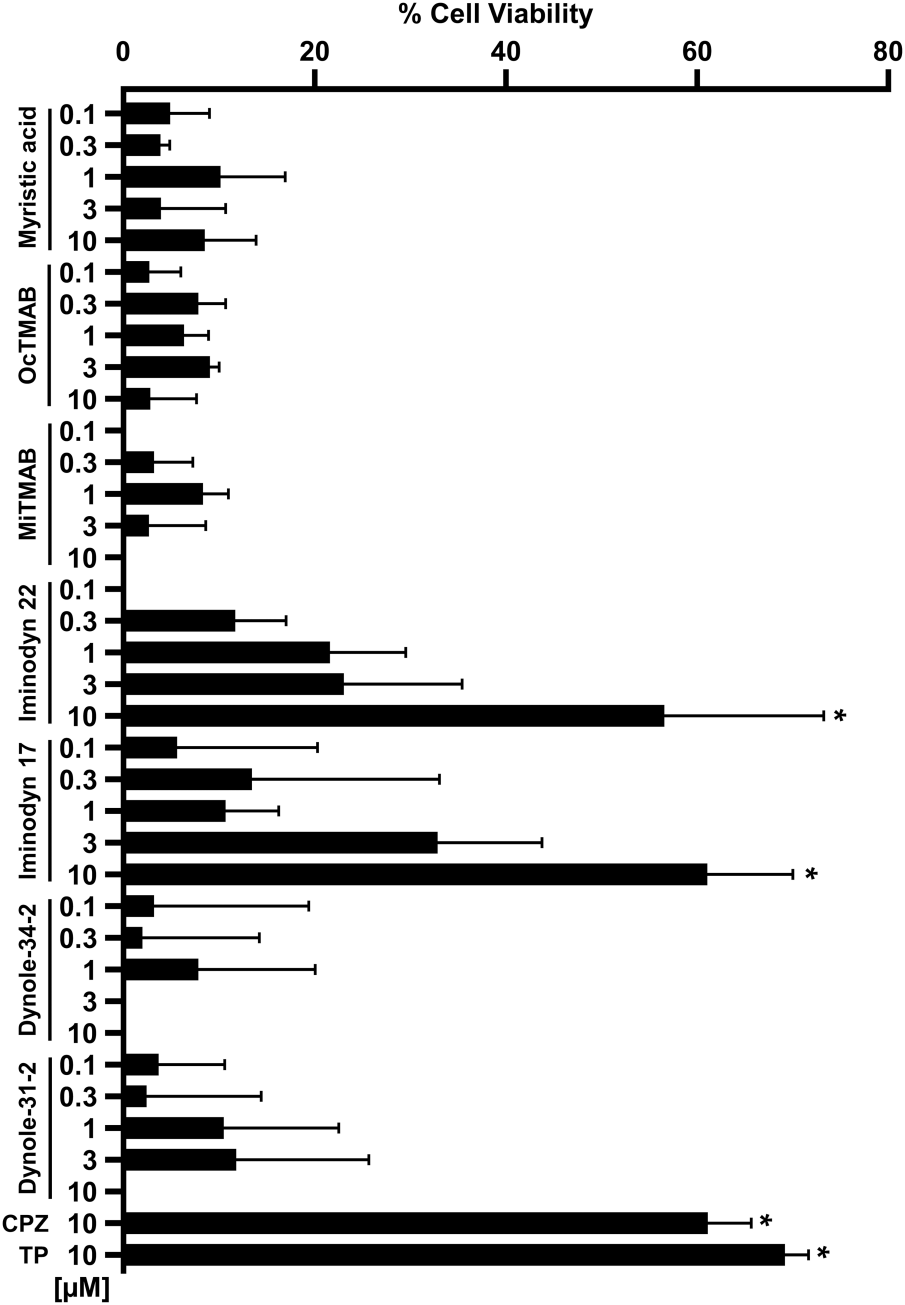
Dynamin inhibitors abrogate the drug-hypersensitizing effect of ΔCR PrP. **A.** The DBCA was employed to evaluate the anti-ΔCR PrP effects of the different dynamin inhibitors. The bar graph shows the quantification of the dose-dependent rescuing effect of each compound at the indicated concentrations. Mean values were obtained from a minimum of 4 independent experiments (n = 4), and expressed as percentage of cell viability rescue. Statistically-significant differences (*) between compound-treated and untreated cells were estimated by Student *t*-test: [Iminodyn-17, 10 μM], *p* = 0,00487; [Iminodyn-22, 10 μM], *p* = 0,00075.

**Fig 10:**
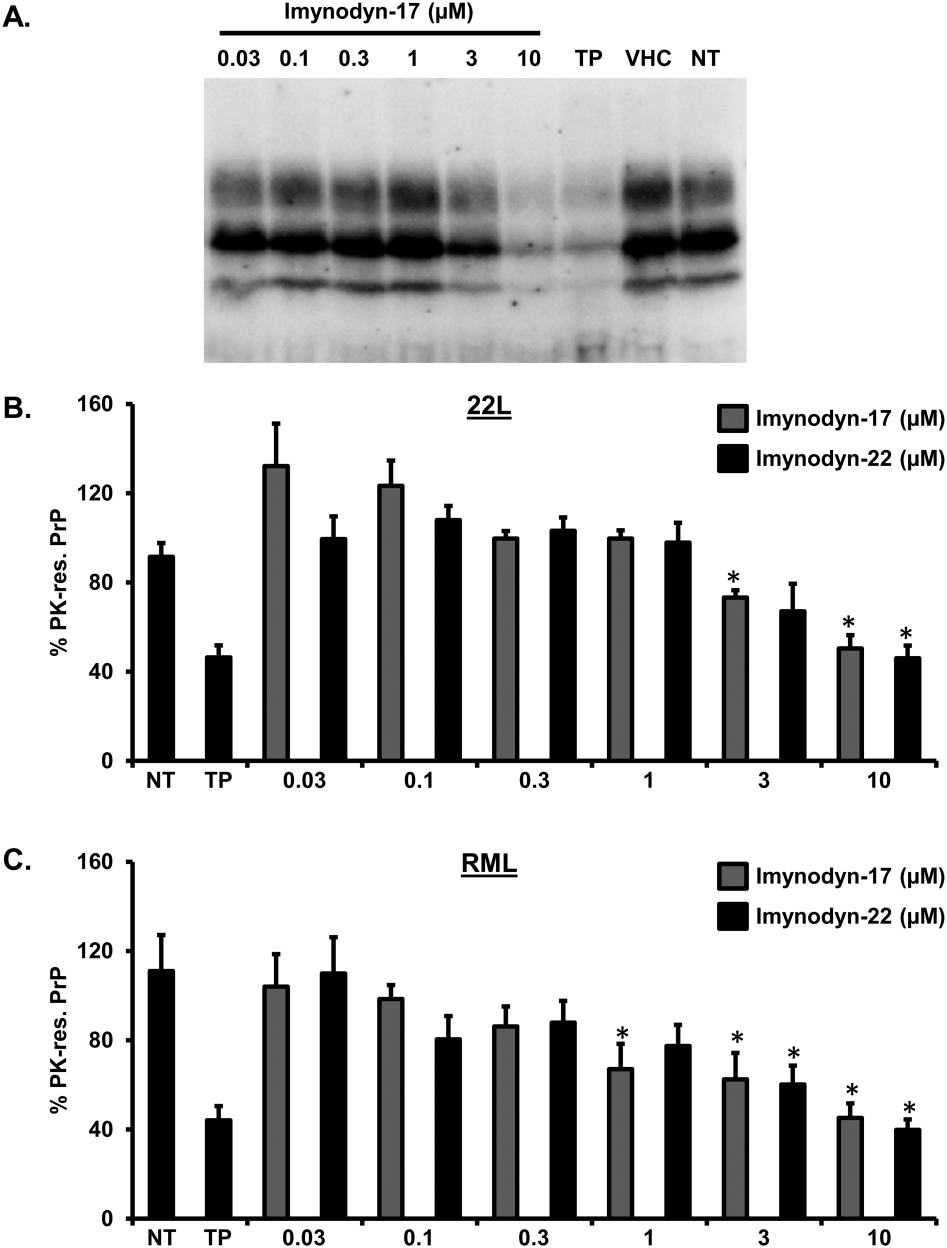
Dynamin inhibitors block the replication of two different mouse prion strains. **A.** N2a cells chronically infected with the 22L prion strain were incubated with increasing concentrations (indicated) of Iminodyn-17 or Iminodyn-22 for 72h, using untreated (Unt.) or vehicle (VHC)-treated cells as negative controls, and Fe(III)-TMPyP as positive control. PK-resistant PrP levels were quantified by Western blotting using anti-PrP^C^ antibody D18. **B-C.** The levels of PK-resistant PrP in RML-(B) or 22L-(C) infected N2a cells after treatment with dynamin inhibitors were quantified by densitometric analysis of gel blots, signals were normalized on the total amount of proteins (obtained by Ponceau staining) in PK-untreated replicates, and expressed as mean % of the signal in DMSO-treated cells (± standard error). * Statistical differences between dynamin inhibitors and untreated controls were estimated by Student *t*-test. For the 22L strain: [Iminodyn-17, 3 μM], *p* = 0.019671; [Iminodyn-17, 10 μM], p = 0,000685; [Iminodyn-22, 10 μM], *p* = 0,000317. For the RML strain: [Iminodyn-17, 1 μM], *p* = 0.034828; [Iminodyn-17, 3 μM], *p* = 0.026235; [Iminodyn-17, 10 μM], p = 0,009796; [Iminodyn-22, 3 μM], *p* = 0,021711; [Iminodyn-22, 10 μM], *p* = 0,009508.

Based on the observation that pharmacological inhibitors of the GTPase domain of dynamin I and II can induce a CPZ-like redistribution of EGFP-PrP^C^ from the cell surface, we sought to test whether these compounds can also reproduce the pharmacological effects of CPZ. First, we employed the DBCA. HEK293 cells stably transfected with ΔCR PrP were exposed to Zeocin (500 μg/mL) for 48h, in presence (0.1–10 μM) or absence of each of the seven dynamin inhibitors. Interestingly, we found that the two compounds (Iminodyn-17 and Iminodyn-22) most effective in inducing the relocalization of EGFP-PrP^C^ from the cell surface, were also the most active against the drug-hypersensitizing effects of mutant PrP (Fig 9). Next, we tested the ability of these molecules to inhibit the replication of two different prion strains in cell cultures. We exposed N2a cells chronically infected with the 22L or the RML mouse prion strains to different concentrations of Iminodyn-17 or Iminodyn-22 (0.03–10 μM) for 72h. Consistent with the results obtained above, the two compounds showed an inhibitory effect against prion replication at concentrations as low as 3 μM (Fig 10). Importantly, the molecules were active against both prion strains, supporting the notion that removing PrP^C^ from the cell surface may inhibit prion propagation in a strain-independent fashion.

## Discussion

CPZ, an FDA-approved phenothiazine analogue used as antipsychotic for its dopamine-antagonist properties, was originally reported to prolong lifespan in mice intracerebrally infected with a mouse prion [45], and later shown to inhibit prion propagation in cell cultures [6]. By employing NMR and X-ray crystallography, a recent study identified a direct interaction between CPZ and PrP^C^ [30]. In this study, we sought to characterize the anti-prion effects of CPZ in various experimental contexts, and investigated further its mechanism of action. We confirm that CPZ inhibits prion propagation in cell cultures, and show that the molecule also suppresses the cytotoxic effects of a PrP mutant. However, we also demonstrate that CPZ is unable to inhibit prion replication in vitro, and provide direct experimental evidence indicating that the compound fails to bind PrP^C^ at biologically-relevant concentrations. Instead, we find that CPZ likely exerts biological effects by inducing the redistribution of PrP^C^ from the cell surface to intracellular compartments. Interestingly, this effect could be mimicked by two specific inhibitors of the GTPase domain of dynamins, proteins involved in the metabolism of membrane vesicles, which were recently suggested to be a pharmacological target for CPZ [39]. Consistent with these data, we show that dynamin inhibitors block prion replication and mutant PrP toxicity in cell cultures.

### CPZ does not exert anti-prion effects by directly binding to PrP^C^

Despite CPZ was known to exert anti-prion effects more than three decades ago, its mechanism of action was not postulated until recently, when a biophysical characterization of two phenothiazines (promazine and CPZ) bound to recombinant PrP^C^ was reported [30]. The binding site of these molecules on PrP^C^ was identified in a hydrophobic pocket made by residues from the two anti-parallel β-sheets (β1 & β2) and the second helix (α2). Interestingly, the study also described an unexpected allosteric effect induced by the interaction of phenothiazines to PrP^C^, leading to the refolding of the unstructured region proximal to β1 onto the C-terminal, globular domain of the protein. These data suggested a precise mechanism by which CPZ stabilizes the native folding of PrP^C^ (i.e. allosteric pharmacological chaperone) [23]. Our data call into question the biological relevance of these conclusions, for at least two reasons. First, if CPZ truly acts as a pharmacological chaperone for PrP^C^, then it should be capable of inhibiting prion propagation both in cells and in vitro. This property was previously confirmed for another PrP^C^ ligand, the porphyrin Fe(III)-TMPyP. Conversely, we found that CPZ fails to inhibit prion amplification in the PMCA reaction. Second, if the anti-prion mechanism of CPZ depends on a direct interaction with PrP^C^, then the affinity of the compound for its target should theoretically be within the same concentration range of its IC_50_ in biological assays. Unfortunately, an affinity value for the binding of CPZ to PrP^C^ was not reported in the original study. Here, we employed two complementary biophysical techniques, SPR and DMR, previously used to define the binding of Fe(III)-TMPyP to PrP^C^, and found that CPZ interacts weakly with PrP^C^, with an estimated K_D_ higher than 400 μM. This result is compatible with data collected in the study from Baral et al. [30], which employed millimolar concentrations of CPZ to carry out NMR and X-ray crystallography analyses. However, CPZ showed anti-prion effects in cells at concentrations approximately two orders of magnitude lower (we estimated the IC_50_ against the replication of the 22L prion in N2a cells as 4.28 μM, fully compatible with previous reports). Such discrepancy clearly indicates that the anti-prion effects exerted by CPZ are not dependent upon the direct binding to PrP^C^.

### CPZ redistributes PrP^C^ from the cell surface

CPZ belongs to the class of phenothiazine derivatives, known as typical antipsychotics, which act by blocking the D2 family of dopamine receptors at nanomolar concentrations [46]. However, many of these compounds have also been known for long time as potent inhibitors of CME, when dosed at low μM concentrations [47]. In fact, CPZ is widely used as a research tool to suppress endocytosis of specific receptors or viruses [40,48]. However, despite the deal of evidence, the precise mechanism by which CPZ inhibits CME is not clear. Previous studies reported that CPZ inhibits the recycling of different receptors by altering the function of the AP2 adaptor, a multimeric complex acting as a cargo in the clathrin-mediated internalization of membrane vescicles [49]. For example, CPZ was shown to alter the cell surface levels of the LDL receptor, causing its relocation in endosomal sites [50]. These data are compatible with our own observations regarding the redistribution of PrP^C^ from the cell surface to intracellular compartments after treatment with CPZ, an effect that we demonstrated using both EGFP-tagged and untagged PrP^C^ molecules, in two different cell types (N2a and HEK293 cells). Importantly, the concentration at which CPZ causes PrP^C^ redistribution from the cell surface (between 3 and 10 μM) perfectly correlates with the observed anti-prion effects. Another recent work suggested that CPZ may act by inducing the redistribution of PrP^Sc^ from organelles in the endocytic-recycling pathway to late endosomes/lysosomes, prior to relocalization of PrP^C^ [51]. This conclusion is partially compatible with our results, since it is likely that altering the localization of PrP^C^ during prion infection may also lead to a change in the distribution of PrP^Sc^. However, our data indicate a primary effect of CPZ on PrP^C^ localization, as the compound suppresses the hypersensitivity to cationic antibiotics conferred by the expression of non-infectious, non-aggregated ΔCR PrP, an effect which was previously demonstrated to critically depend on the presence of the mutant molecules at the plasma membrane [52].

### The effects of CPZ are mimicked by inhibitors of the GTPase domain of dynamins

A previously documented effect of phenothiazines, including CPZ, is the inhibition of large GTPase dynamins [39]. In a recent study, several phenothiazine-derived molecules were evaluated for their ability to inhibit dynamin GTPase activity and CME. The authors reported a robust correlation between dynamin and CME inhibition for several compounds, suggesting that dynamins are common targets of CME-inhibiting phenothiazines. In light of these data, we sought to test whether inhibition of dynamins could reproduce the effects of CPZ on PrP^C^ distribution. Our analyses led to at least two important observations. First, we collected evidence that well-characterized inhibitors of the GTPase domain of dynamin I and II alter the cell surface localization of PrP^C^ in a CPZ-like manner. These data are consistent with previous reports suggesting a role for dynamins in the recycling of PrP^C^ from the plasma membrane [42,53]. Dynamins are known to play a role in the scission of membrane vescicles at different levels along both the exocytic and endocytic pathways [54]. Thus, inhibiting these factors may lead to the retention of PrP^C^ in the secretory pathway, and/or its accumulation in membrane-attached endosomal vesicles, in both cases causing an overall decrease of PrP^C^ at the cell surface. Second, we demonstrated that dynamin inhibitors block the replication of two different mouse prions in cell cultures and, similarly to CPZ, also abrogate the toxic effect of mutant ΔCR PrP. These results provide insights into the role of dynamins in the intracellular trafficking of PrP^C^, with potential relevance for targeting these proteins in prion diseases.

### Removing PrP^C^ from the cell surface as a therapeutic strategy for prion diseases

A great deal of data support the notion that prion conversion requires the expression of PrP^C^ at the cell surface. In fact, genetic or enzymatic depletion of PrP^C^ from the plasma membrane has been shown to block prion replication in cell cultures and in vivo [55–57]. In addition, the absence of PrP^C^ at the neuronal surface has been reported to abrogate the neurotoxicity delivered by PrP^Sc^ generated in surrounding astrocytes [21]. Thus, removing PrP^C^ from the cell surface may produce inhibitory effects on prion infectivity and toxicity. In support of this conclusion, a number of lipid-interacting or cholesterol-lowering compounds have been described to act as anti-prion compounds by altering the cellular distribution of PrP^C^ [5]. Our study adds to this list CPZ, and at least two dynamin inhibitors (Iminodyn-17 and Iminodyn-22). Unfortunately, a relevant problem affecting all these molecules is their specificity [47]. We observed that the effects on PrP^C^ localization were obtained at concentrations only slightly lower than those causing cytotoxicity. This evidence may likely reflect an intrinsic propensity of CPZ and dynamin inhibitors to induce the relocalization of multiple surface proteins, ultimately affecting cell viability. Future efforts aimed at identifying drug-like molecules capable of selectively removing PrP^C^ from the cell surface could lead to the identification of novel and effective compounds against prion diseases.

## Conclusions

In summary, we have shown that PrP^C^ is not the direct pharmacological target of CPZ. Instead, this compound likely blocks prion replication by altering the cell surface distribution of PrP^C^, an effect also produced by specific inhibitors of the GTPase activity of dynamins. Consistently, we report that dynamin inhibitors also block the replication of two different prion strains in cell cultures, and abrogate the cytotoxic effects of a PrP mutant. In addition to clarify the mechanism of action of CPZ, this study suggests that dynamins may represent novel pharmacological targets for prion diseases.

## Materials and methods

### Ethics statement

All the experiments involving animals adhered to the guidelines contained in the Italian Legislative Decree 116/92, which transposed the European Directive 86/609/EEC on Laboratory Animal Protection, and then in the Legislative Decree 26/2014, which transposed the European Directive 2010/63/UE on Laboratory Animal Protection. The research protocol was performed under the supervision of the Service for Biotechnology and AnimalWelfare of the Istituto Superiore di Sanità, and was approved by the Italian Ministry of Health (decree number 84/12.B).

### Prion-infected cells

A sub-clone of mouse N2a cells previously selected as highly susceptible for prion infection (called N2a.3) were grown in culturing medium [Dulbecco’s Minimal Essential Media (DMEM), 10% heat-inactivated fetal bovine serum (Δ56-FBS), Penicillin/Streptomycin (Pen/Strep) and non-essential amino acids (NEAA)], and passaged 5–7 times after infection with the 22L or RML prion strains, both derived from corresponding prion-infected mice (brain homogenates were kindly provided by Dr. Roberto Chiesa, Mario Negri Institute for Pharmacological Research, Milan, Italy). In order to test the anti-prion effects of CPZ (Sigma Aldrich) or dynamin inhibitors (Abcam, Bristol, UK), cells were seeded in 24-well plates (day 1) at approximately 60% confluence, with different concentrations of each compound, or vehicle control (DMSO). Medium containing fresh compounds or vehicle was replaced on day 2, and cells were then split (1:2) on day 3, avoiding the use of trypsin by pipetting directly onto the well surface. Cells were collected on day 4 by adding 500 μL of Phosphate-buffered saline (PBS) directly into each well. Cell pellets where obtained by centrifuging at 3.500 rpm x 3 min, and then rapidly stored -80°C. In order to estimate the amount of PK-resistant PrP molecules, cell pellets were resuspended in 20 μL of lysis buffer (PBS, pH 7.4, 0.5% NP-40, 0.5% TX-100) and incubated for 10 min at 37°C with 2,000 units/mL of DNase I (New England Biolabs, UK). Half of the resulting sample was incubated with 10 μg/mL of PK for 1h at 37°C, while the other half was incubated in the same conditions in absence of PK. Both PK-treated and untreated samples were then mixed with 4X Laemmli sample buffer (LMSB; 2% SDS, 10% glycerol, 100 mM Tris-HCl pH 6.8, 0.002% bromophenol blue, 100 mM DTT), boiled for 10 min at 95°C in a thermomixer, and ran in SDS-PAGE. The total amount of proteins in PK-untreated samples were then visualized by Ponceau staining (Sigma Aldrich), while PK-treated samples were analyzed by Western blotting.

### Western blotting

Analyses by Western blotting were performed as previously described [44,58]. Briefly, samples were loaded on SDS-PAGE carried out using 12% acrylamide pre-cast gels (BioRad, CA, USA). Separated proteins were electrophoretically transferred to polyvinylidene fluoride (PVDF) membranes (Thermo Fisher Scientific, MA, USA) which were then blocked for 20 min in 5% (w/v) non-fat dry milk in Tris-buffered saline containing 0.05% Tween-20. Blots were probed with anti-PrP antibody D18 (1:1000), followed by goat anti-human IgG conjugated with horseradish peroxidase (Santa Cruz Biotechnology, CA, USA). Signals were revealed using the ECL Prime Western Blotting Detection Kit (GE Healthcare, UK), and visualized with a ChemiDoc Touch Imaging System (Bio-Rad, CA, USA).

### PMCA

All brain tissue samples employed in these analyses were collected firsthand from the animals "naturally" infected with prion strains. Two different PMCA protocols were employed in this study, as previously described [25,32,33]. In the first one (Fig 2A), 2–3 month-old bank voles homozygous for methionine at codon 109 (Bv109M) were used as substrate for the reaction. Vole brains were perfused and quickly homogenized in conversion buffer (PBS 1X, pH 7,4; 0.15 M NaCl; 1% Triton X) containing mini-Complete protease inhibitor (Roche; 10% w/v), and then stored at -80°C. CPZ (final concentration 100–500 μM), Fe(III)-TMPyP (100 μM) or vehicle control (an equivalent volume of distilled water) were added to the substrate just prior to each PMCA experiment. Seeds were prepared using brain tissue from Bv109M terminally affected with a Bv109M-adapted prion strain derived from the Italian sheep PrP^Sc^ isolate SS7. Brain tissues dissected from terminally ill mice were homogenized in PBS (10% w/v), diluted 1:10 or 1:100 in PMCA substrate, immediately subjected to PMCA, or frozen at -20°C. Samples were subjected to 32 continuous sonication/incubation cycles (20 sec pulse at 80% power, followed by incubation for 30 min at 37°C), using a Misonix S3000 plate sonicator. Amplified and frozen samples were digested with PK, and PrP signals analyzed by Western Blotting. Blots were probed with anti-PrP monoclonal antibody SAF84 (a.a. 167–173 Bank vole PrP sequence; 1.2 μg/mL), followed by HRP-conjugated anti-mouse immunoglobulin (Pierce). Amplification factor was calculated by quantifying the amount of PK-resistant PrP in post-PMCA 1:100 diluted samples (Y) and in the 1:10 frozen dilution (X), using the formula (Y/X) x 10. Results were then expressed as the mean value (± standard deviation) of at least 4 independent samples. For the second protocol (Fig 2B and 2C), infected brain homogenates (10–1 in PBS) to use as seed for PMCA were prepared manually using a glazed mortar and pestle from a brain of sheep clinically affected by Scrapie (Dawson isolate) supplied by the Ecole Nationale Vétérinaire de Toulouse (INRA). The in vitro prion amplification and PK-resistant PrP detection of amplified samples were performed as described previously with minor modifications. Briefly, Tg338 brains used for substrate were perfused using PBS + 5 mM EDTA and the blood-depleted brains were frozen immediately until required. A 50 μl aliquot of 10% Tg338 brain homogenate, seeded with different dilutions of scrapie (Dawson isolate) were loaded onto 0.2-ml PCR tubes. Samples were added 0.5 μl of DMSO, TMPyP-Fe(III) (used as positive control at final concentration 100 and 10 μM) and Chlorpromazine diluted in DMSO (at final concentration of 500, 100 and 10 μM), and placed into a sonicating water bath at 37–38°C without shaking. Tubes were positioned on an adaptor placed on the plate holder of the sonicator (model S-700MPX, QSonica, Newtown, CT, USA) and subjected to a unique 48 h round of incubation cycles of 30 min followed by a 20 s pulse of 150–220 watts sonication at 70–90% of amplitude. In order to detect PK-resistant PrP species, PMCA treated samples were incubated with 200 μg/ml of PK for 1 h at 42°C with shaking (450 rpm). Digestion was stopped by adding electrophoresis Laemmli loading buffer and the samples were analyzed by Western blotting.

### Plasmids

Cloning strategies used to generate the cDNAs encoding WT, Δ105–125 (ΔCR), or EGFP-tagged PrP have been described elsewhere [59,60]. The EGFP-PrP construct contains a monomerized version of EGFP inserted after codon 34 of mouse PrP^C^. The identity of all constructs was confirmed by sequencing the entire coding region. All constructs were cloned into the pcDNA3.1(+)/hygro expression plasmid (Invitrogen).

### DBCA

The DBCA protocol employed in this study was performed as described previously [37,38]. Briefly, HEK293 cells (ATCC, CRL-1573) expressing ΔCR PrP were seeded on 24-well plates at approximately ~60% confluence (day 1). On day 2, cells were treated with 500 μg/mL of Zeocin, and with different concentrations of CPZ, Fe(III)-TMPyP, dynamin inhibitors or vehicle controls (volume equivalent). Medium containing fresh antibiotic/compounds was replaced daily until day 3, after which the medium was removed, and cells were incubated with 1 mg/mL of 3-(4,5-dimethylthiazol-2-yl)-2,5-diphenyltetrazolium bromide (MTT, Sigma Aldrich, St. Louis, MO) in PBS for 30 min at 37°C. After carefully removing MTT, cells were resuspended in 500 μL of DMSO, and cell viability values obtained by a plate spectrophotometer (BioTek Instruments, VT, USA), measuring absorbance at 570 nm.

### SPR

Interaction studies using SPR were conducted on a ProteOn XPR36 Protein Interaction Array system (Bio-Rad), as previously reported [25,61]. Approximately 16,000 resonance units (RUs) of full-length mouse or human recombinant PrP were immobilized on the surface of a SPR chip (GL-H chip, Bio-Rad) by an amine-coupling reaction. After a stabilization and washing of the chip surface, different concentrations of CPZ (up to 100 μM, diluted in PBS/0.05%, Tween-20) were perfused for 130 sec to monitor association, followed by a buffer wash to allow dissociation. CPZ-specific signals were obtained by subtracting reference channels (where no protein was immobilized, or only buffer injected). The resulting sensorgrams (time course of resonance unit signals) did not allow data fitting, being the intensity of the signals too low, or the association/dissociation curves too steep, reflecting a low affinity interaction.

### DMR

The detection of binding events at the equilibrium was performed using the label free, DMR module of an EnSight Multimode Plate Reader (Perkin Elmer, MA, USA) [25]. Mouse or human recombinant PrP were immobilized onto the surface of DMR plates (15 μL/well of a 2.5 μM recombinant PrP solution in 10 mM sodium acetate buffer, pH 5) using an amine-coupling chemistry. The interaction between CPZ and recombinant PrP or BSA was evaluated by incubating different concentrations of the compound (0.1–2,000 μM; diluted in assay buffer: 10 mM PO4, pH 7.5, 2.4 mM KCl, 138 mM NaCl, 0.05% Tween-20) for 30 min at room temperature. All steps were performed by using a Zephyr Compact Liquid Handling Workstation (Perkin Elmer). Final signals were obtained by automatic intra-well, empty surface normalization, and by subtraction of the control wells (no protein immobilized, or vehicle added). The Kaleido software (Perkin Elmer) was used to acquire and process the data.

### Immunostaining

Cells were seeded on glass coverslips coated with Poly-L-lysine (0.05 mg/ml) and grown for 24 h to 60% confluence. For surface staining of PrP, cells were incubated for 15 min at 4°C with D18 antibody diluted 1:300 in Opti-MEM (Life Technologies, Inc.), followed by washing with PBS and fixation in 4% paraformaldehyde for 30 min at 4°C. Coverslips were then washed with PBS, incubated with blocking solution (2% FBS in PBS) for 30 min at RT and then with Alexa 488-conjugated goat anti-human IgG (Invitrogen) diluted 1:500 in blocking solution. For total PrP staining, cells grown on glass coverslips were washed with PBS and fixed for 30 min at 4°C with 4% paraformaldehyde in PBS. Cells were then washed with PBS, permeabilized with Triton X-100 (Sigma) 0.1% in PBS for 1 min and washed again with PBS. After incubation with blocking solution (2% FBS in PBS), coverslips were incubated for 1h at RT with D18 antibody (1:300 in blocking solution) and after washing incubated with the secondary antibody as performed above. Coverslips were mounted with Fluor-save Reagent (Calbiochem), and analyzed with a Zeiss Imager M2 microscope.

### Cell blotting

The cell blotting assay was employed here as previously described [44], to evaluate the ability of CPZ or Fe(III)-TMPyP to induce the removal of PrP^C^ from the cell surface. N2a cells stably expressing mouse, WT PrP^C^ were grown to confluence on glass coverslips. In order to detect surface PrP^C^, coverslips were incubated at 0°C for 1h with antibody 3F4 (1:1000). This step was omitted for the detection of total PrP^C^. All coverslips were then blotted onto a nitrocellulose membrane soaked in lysis buffer (PBS, pH 7.4, 0.5% NP-40, 0.5% Na- deoxycholate, plus complete EDTA-free Protease Inhibitor Cocktail Tablets, Roche). The portion of the membrane corresponding to coverslips previously exposed to 3F4 was cut and directly incubated with HRP-conjugated, anti-mouse secondary antibody. Conversely, the rest of the membrane, blotted with coverslips used for detecting total PrP^C^, was incubated with both primary (3F4) and secondary antibodies. PrP signals in all the membranes were then revealed by enhanced chemiluminescence (Luminata, BioRad), visualized by a Bio-Rad XRS Chemidoc image scanner (Bio-Rad), and quantified by densitometry (Quantity One software, Bio-Rad). The amount of PrP^C^ on the cell surface was derived from the ratio of surface-to-total PrP^C^ signals.

### Cell imaging

HEK293 cells (ATCC, CCL-2) stably expressing WT or EGFP-PrP were seeded on 8-well chamber slides (Ibidi, Germany) and grown for 24/48h to reach approximately 60% confluence. Cells were then treated with different concentrations of CPZ or Fe(III)-TMPyP at various time points, washed with PBS, and fixed with 4% paraformaldehyde. Coverslips were mounted with a gel mount (Sigma Aldrich), and visualized with a CellR imaging station (Olympus) coupled to an inverted microscope (IX 81, Olympus). The fluorescent signal deriving from EGFP was acquired with a high-resolution camera (ORCA) equipped with a 488 nm excitation filter, and an emission filter with a range of 510 ± 40 nm. In an alternative experimental setting, the cell surface localization of PrP^C^ was monitored using an Operetta High-Content Imaging System (Perkin Elmer, MA, USA). Briefly, cells were plated on CellCarrierUltra 384 plates (PerkinElmer) and grown for 24h to obtain a confluent layer. Cells were then treated with varying concentrations of drugs for 24h. Plates were fixed with 4% paraformaldehyde (Thermo) and counterstained with Hoechst 33342 (Thermo). Imaging was performed using a 20X High NA objective. Five fields were acquired in each well over two channels (380–445 Excitation-Emission for Hoechst and 475–525 for EGFP). Imaging analysis was performed using the Harmony software version 4.1 (Perkin Elmer). Image segmentation consisted of two key steps: nuclei identification by the Hoechst signal, and selection of the regions of interest based on the EGFP signal. The average fluorescence intensity of the EGFP channel was picked in the membrane region (enlarged border of the cell), as well the region inside the cell. As a measure of the degree of PrP internalization from the plasma membrane to intracellular compartments, the membrane/cellular (M/C) fluorescence intensity ratio was calculated for each cell. To better discriminate PrP internalization, the threshold of M/C = 1.5 was inferred as the median value of the control cells in different experiments (data not shown). The fraction of cells showing a M/C>1.5 was estimated for each condition and normalized to the control samples (% surface/internal EGFP PrP). Cytotoxicity was quantified by counting the cell nuclei, and expressed as the percentage of reduction of cells after each treatment.

## Supporting information

S1 Fig Intrinsic cytotoxicity of CPZ. HEK293 cells stably expressing WT (A) or EGFP- (B) PrP were treated with increasing concentrations of CPZ (0.3–10 μM), TP (10 μM) or vehicle (VCH) controls for 3 48 h. Cell viability was then estimated by MTT assay. Bars represent mean values of four (n = 4)independent experiments (± standard error). No statistical differences were detected between the 5 samples.
(TIF)

S2 Fig Effect of myristic acid on the distribution of EGFP-PrP^C^. HEK293 cells stably expressing EGFP-PrP^C^ were grown to ~80% confluence on 384-well plates, and then incubated with the indicated concentrations of each compound. **A.** Chemical structure of each myristic acid, and representative images. **B.** The graph shows the mean percentage of cells (± standard deviation) presenting a ratio of surface vs intracellular EGFP-PrP^C^ signal higher than 1.5. **C.** The graph shows the mean percentage (± standard deviation) of the total number of nuclei detected in each well by Hoechst staining.
(TIF)

S3 Fig Effect of MiTMAB on the distribution of EGFP-PrP^C^. **A.** Chemical structure of MiTMAB, and representative images. **B.** The graph shows the mean percentage of cells (± standard deviation) presenting a ratio of surface vs intracellular EGFP-PrP^C^ signal higher than 1.5. **C.** The graph shows the mean percentage (± standard deviation) of the total number of nuclei detected in each well by Hoechst staining.
(TIF)

S4 Fig Effect of OcTMAB on the distribution of EGFP-PrP^C^. **A.** Chemical structure of OcTMAB, and representative images. **B.** The graphs show the mean percentage of cells (± standard deviation) presenting a ratio of surface vs intracellular EGFP-PrP^C^ signal higher than 1.5. **C.** The graphs show the mean percentage (± standard deviation) of the total number of nuclei detected in each well by Hoechst staining.
(TIF)

S5 Fig Effect of Dynole-31-2 on the distribution of EGFP-PrP^C^. **A.** Chemical structure of Dynole-31-2, and representative images. **B.** The graphs show the mean percentage of cells (± standard deviation) presenting a ratio of surface vs intracellular EGFP-PrPC signal higher than 1.5. C. The graphs show the mean percentage (± standard deviation) of the total number of nuclei detected in each well by Hoechst staining.
(TIF)

S6 Fig Effect of Dynole-34-2 on the distribution of EGFP-PrP^C^. **A.** Chemical structure of Dynole-34-2, and representative images. **B.** The graphs show the mean percentage of cells (± standard deviation) presenting a ratio of surface vs intracellular EGFP-PrPC signal higher than 1.5. C. The graphs show the mean percentage (± standard deviation) of the total number of nuclei detected in each well by Hoechst staining.
(TIF)

S7 Fig Example of quantification of membrane vs intracellular EGFP-PrP. Cells treated with vehicle (A-C) or CPZ (20μM, D-F) for 24h were fixed and counterstained with Hoechst. Images were acquired by detecting Hoechst-stained cell nuclei (380-445nm excitation-emission) as well the intrinsic EGFP fluorescence (and 475-525nm). The average fluorescence intensity of EGFP corresponding to the membrane region (enlarged edge of the cell) was then compared to the intracellular EGFP signal. PrP internalization was then detected by quantifying the membrane/cellular (M/C) ratio, and expressed as the % of cells showing a M/C>1.5 (panels C and F).
(TIF)
