# Supplementary figures and images for: An antipsychotic drug exerts anti-prion effects by altering the localization of the cellular prion protein
Source: PLoS One. 2017 Aug 7;12(8):e0182589. doi: 10.1371/journal.pone.0182589 (PMC5546605; doi:10.1371/journal.pone.0182589)

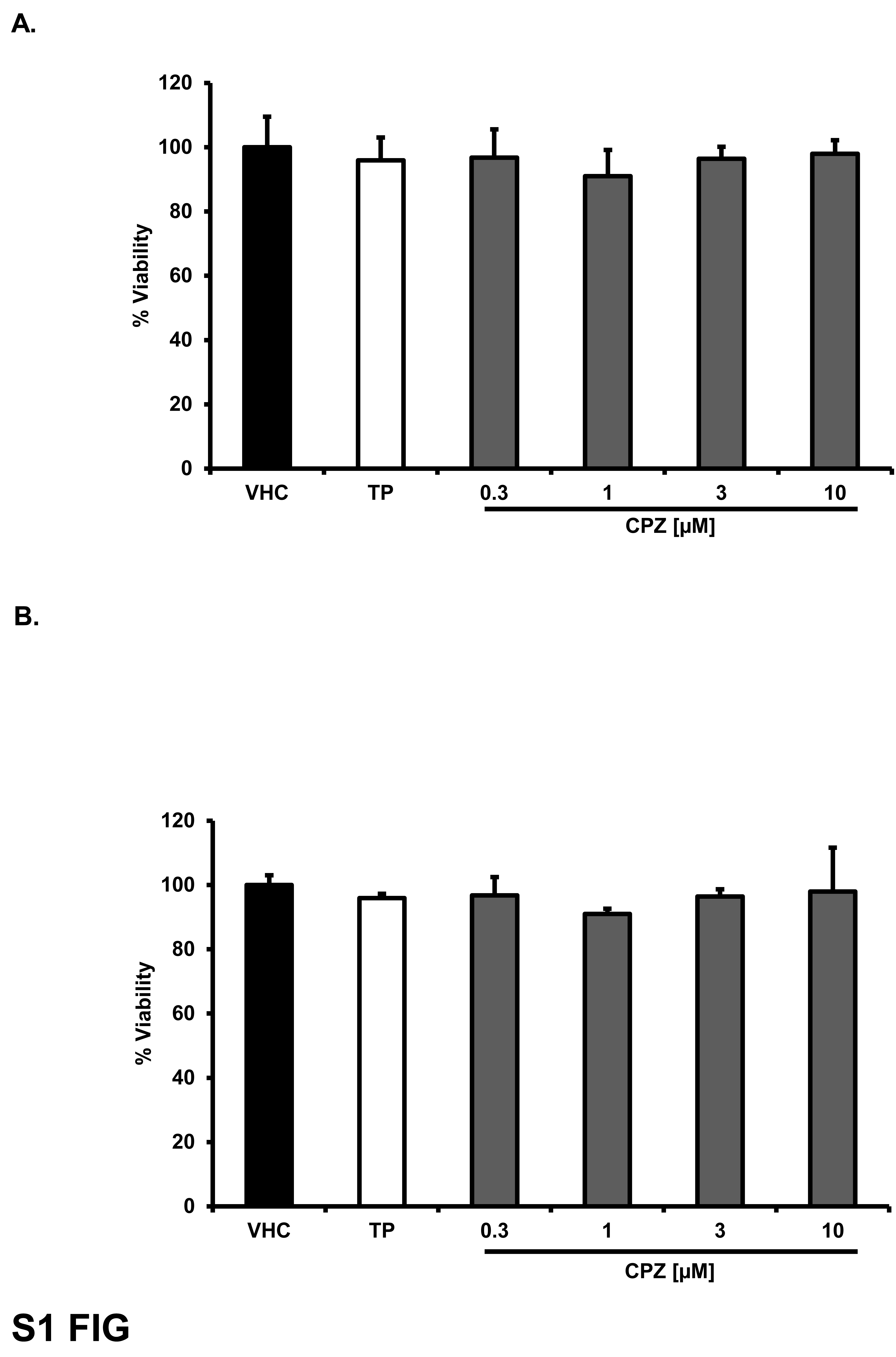

Supplement: S1 Fig — HEK293 cells stably expressing WT (A) or EGFP- (B) PrP were treated with increasing concentrations of CPZ (0.3–10 μM), TP (10 μM) or vehicle (VCH) controls for 3 48 h. Cell viability was then estimated by MTT assay. Bars represent mean values of four (n = 4)independent experiments (± standard error). No statistical differences were detected between the 5 samples. (TIF) [file pone.0182589.s001.tif]
